# London's Institutional Medical Relief: Proposals for Its Reform

**Published:** 1918-04-13

**Authors:** C. Thackray Parsons


### LONDON'S INSTITUTIONAL MEDICAL RELIEF.

### PROPOSALS FOR ITS REFORM.

By C. THACKRAY PARSONS, M.D.,Lond.

Poor-Law Reform has again been brought prominently to notice by the Report of the Local Government Board Committee of the Ministry of Reconstruction. Those who have advocated the reform of the present system of administration of the Poor Laws approve in general of the reforms suggested in the Report. The Committee recommended the transference of all the institutions administered by Boards of Guardians, by the Managers of Sick Asylums and Poor-Law School Districts in London, and by the Metropolitan Asylums Board to the London County Council. I wish, in this paper, to draw attention to the improvement in institutional medical relief which could be effected if the suggestion were carried out.

### A Long-needed Reform in Sight.

At present, with some exceptions, such, for instance, as the important work carried out by the Metropolitan Asylums Board, each Poor-Law district makes its own arrangements for institutional medical relief without any consideration of the arrangements made in adjoining areas. The result is that each district has its own infirmary, its own mixed workhouse, its own institution for children, and in some cases, also, institutions for special cases, which may or may not receive boarders from other parishes. As regards the infirmaries, the standard of equipment varies greatly. Some are built in accordance with the requirements of modern hospital construction and are well equipped, others are old buildings not suited for the treatment of acute cases, or for operative surgical work, and are often insufficiently equipped. None of them reaches the standard of equipment and staffing set by the large voluntary hospitals. The expensive provisions for exact diagnosis and for the treatment of special cases made in these hospitals would not be justified in each individual infirmary because the number of cases in any one of them likely to benefit thereby is too small. Another drawback to the present system is that the infirmary of one district may be overcrowded for the greater part of the year, whilst at the same time a neighbouring infirmary is partly empty.

### What Would Follow.

Now if all these institutions were transferred to the management of the County Council the position would be very different. The best of the infirmaries could be set apart for acute medical and surgical cases. There are enough of them to provide accommodation for these cases at convenient points throughout the county area. Each of them should be provided with an operating theatre, with an $x$-ray installation, and with apparatus sufficient to carry out all ordinary medical and surgical work. The infirmaries not suitable for dealing with these acute cases, and some of the present workhouses, should be allotted for the reception of chronic cases and cases requiring less elaborate treatment. Other workhouses, again, would make excellent homes for the aged, for cripples, and for the incurable. One would expect, too, to see convalescent homes in the country provided. Except as regards the institutions for children of the Metropolitan Asylums Board this important and money-saving provision has been almost entirely neglected by Boards of Guardians.

### The Scheme in Operation.

In order to bring the scheme of medical relief up to the level of the large voluntary hospitals further special accommodation is required. One institution would have to be adapted to form a central laboratory and to provide the more elaborate and costly forms of special treatment. It would have at least the following departments: (1) a complete $x$-ray installation capable of taking instantaneous photographs and of being used for the more difficult forms of $x$-ray diagnosis and for $x$-ray treatment; (2) a department for treatment by light rays, ultra violet rays, diathermy, and high frequency; (3) a department for treatment by radium; (4) a pathological and bacteriological department; (5) a chemical department for the examination of morbid products and for testing drugs, foodstuffs, and other supplies; and (6) a department for the exact diagnosis and study of cardiac conditions. This central laboratory would have to have a few beds for special cases, but most of its patients would live in the various infirmaries, and would be sent to it for examination and treatment. Each department would naturally have its staff of experts, whose services would also be available in consultation at any of the infirmaries. The other special institutions required would include at least the following: (1) An orthopædic institution fitted with baths, electrical and mechanical apparatus, a gymnasium, and a massage department. In addition to ordinary orthopædic cases, this centre would also receive such nervous, arthritic, and cardiac cases as would benefit by graduated exercises, baths, electricity, and massage. It would have beds for resident patients, and would also treat patients sent to it. (2) A hospital for ear, throat, and nose cases; (3) an ophthalmic hospital; (4) a hospital for skin cases, with a special section for venereal disease; (5) a hospital for nervous diseases, which would also receive cases of incipient insanity and suitable cases of mental disease; (6) a hospital for gynæcological cases, which would have a section for dealing with difficult midwifery cases and for the treatment of the abnormalities of pregnancy; (7) institutions for treatment (*a*) of early cases, (*b*) of chronic cases, and (*c*) of advanced cases of phthisis; (8) institutions for children suffering from chronic diseases, where arrangements could be made for their education during treatment; (9) a dental hospital; (10) epileptic colonies; and (11) homes for the mentally

**INSTITUTIONAL MEDICAL RELIEF—(*continued*).**

deficient. For some of these special cases a small building would be sufficient, for others two or more institutions would be necessary, but practically all of them could be provided by adapting existing buildings taken over from the Boards of Guardians. It would, of course, be an essential requirement that each of these specialised buildings should have a staff of experts. The patients for these special institutions would be selected at and sent on from the general infirmaries. The comparatively small number of able-bodied inmates of the present mixed workhouses would be transferred to one or more labour colonies.

The scheme detailed above would provide a satisfactory and efficient system of institutional medical relief in London. It could only be carried out by placing all the present institutions in the London area under one authority. Under any other system of administration its cost would be prohibitive. Over it all there should be a principal medical officer assisted by a consulting physician and a consulting surgeon, whose services should be available in consultation at all the institutions. These three, with the medical heads of specialised departments and of the general infirmaries, should form an advisory committee for the County Council. They should also form a medical research committee for the initiation of lines of research and for the stimulation of such work, for which there is abundant and neglected material in our Poor-Law infirmaries.

Some Advantages of Centralisation.

Centralisation would have other advantages. It would lead, in substitution for the present Poor Law, to the formation of a State or Municipal Medical Service properly graded and paid, with opportunities for promotion by merit, for research work and for the study of special diseases, which would attract medical men of the highest attainments and qualifications. In this connection one would expect facilities to be provided at the central laboratory and at the specialised institutions for men from the general infirmaries to work and study in them. It would lead to the development of a uniform system of training for the probationers in the institutions. Overcrowding in individual infirmaries would be stopped. A central department, staffed by experts, for the provision of food, drugs, and necessaries, and for the manufacture of surgical and medical appliances and of hospital furniture, could be established—a reform which would save money and would provide goods of better quality than are obtained by the present system of local contracts. The engineering, building, laundry, and other lay work in the institutions could be supervised by experts with advantage both from the view of economy and of efficiency. A systematic collection of information relating to different methods of administration could be made and would be of the greatest value. A central ambulance system would be more efficient and economical. Lastly, the preventive work of the public health department of the Council would be brought into co-operation with the remedial work carried out in the Council's institutions, and the department for medical statistics could be extended to deal with the results of the clinical work. There is one point in the Committee's recommendations for the reform of medical relief with which I disagree. The Committee suggest that medical assistance in the home should be given by the staff of the medical officer of health of the Borough Council. I think it would be better that this assistance should be given by medical practitioners working under the medical superintendents of the general infirmaries. This would ensure a closer co-operation between the indoor and outdoor work, it would facilitate the selection of cases suitable for indoor treatment, it would enable the outdoor medical man to keep in touch with his cases after their admission to the infirmary, and would make it easier to discharge cases sufficiently recovered to go home but still needing medical supervision and treatment. It is obviously of advantage that there should be a close co-operation between the indoor and outdoor medical officers, and this will be lost if the one officer is working under the County Council and the other under the Borough Council.

